# Case Report: Staged surgical management in ESRD: off-pump CABG followed by renal transplantation to enhance graft survival

**DOI:** 10.3389/fcvm.2025.1486771

**Published:** 2025-02-07

**Authors:** Özge Çetinarslan, Davit Saba

**Affiliations:** ^1^ Department of Cardiology, T.C. Demiroglu Science University, Istanbul Florence Nightingale Hospital, Istanbul, Türkiye; ^2^ Department of Cardiovascular Surgery, T.C. Demiroglu Science University, Istanbul Florence Nightingale Hospital, Istanbul, Türkiye

**Keywords:** end-stage renal disease (ESRD), off-pump coronary artery bypass grafting (OPCAB), on-pump coronary artery bypass grafting (ONCAB), renal transplantation (RT), staged surgery vs. combined surgery

## Abstract

Patients with end-stage renal disease face a significantly higher risk of cardiovascular diseases. For patients who are candidates for renal transplantation (RT), major surgeries such as coronary artery bypass grafting (CABG) are associated with cardiac complications as well as higher rates of post-operative complications, including the need for large amounts of blood transfusion, worsening kidney function, infection, and graft rejection. Studies have shown that blood transfusions can increase the risk of graft rejection due to immune system activation. Off-pump CABG (OPCAB), also known as beating heart surgery, is a technique in which a heart–lung machine is not used, and the heart continues to beat throughout the procedure. The main advantage of OPCAB surgery compared to on-pump CABG (ONCAB) is that it requires fewer blood product transfusions and has fewer renal, pulmonary, and hematological complications. This case series uniquely discusses two patients who underwent successful beating heart CABG without blood transfusion, followed by RT.

## Introduction

Patients with end-stage renal disease (ESRD) face a significantly higher risk of cardiovascular diseases (1). In this population, coronary artery disease (CAD) is driven by complex mechanisms. Excessive medial calcinosis, characterized by calcium deposition in the vascular smooth muscle layer, due to chronic inflammation and imbalances in calcium-phosphate metabolism leads to arterial stiffness and impaired vascular compliance, significantly increasing cardiovascular endpoints in this population. The presence of CAD in patients with ESRD often necessitates coronary artery bypass grafting (CABG) prior to renal transplantation (RT). Unfortunately, in patients who are candidates for RT, major surgeries such as CABG are associated with cardiac complications, worsening kidney function, post-operative infections, and graft rejection. Studies have shown that blood transfusions not only complicate post-operative recovery but also increase the risk of graft rejection due to immune system activation (2, 3).

Off-pump CABG (OPCAB), also known as beating heart surgery, is a technique in which the heart–lung machine is not used, and the heart continues to beat throughout the procedure. The main advantage of OPCAB surgery compared to on-pump CABG (ONCAB) is that it requires fewer blood product transfusions and has fewer renal, pulmonary, and hematological complications (4).

This case series uniquely discusses two patients who underwent successful OPCAB without blood transfusion, followed by RT. This case series also aims to provide a perspective on the debate between combined and staged CABG and RT, despite the increasing popularity of combined surgeries. Written informed consent was obtained from all patients.

## Case 1

**Figure 1:**
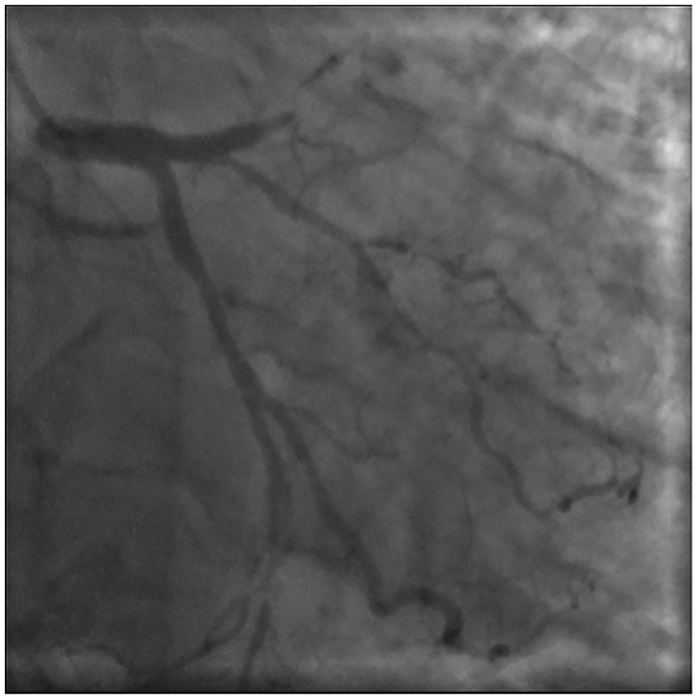
Subtotal occlusion of the LAD and Cx-OM2 artery in a coronary angiogram.

**Figure 2:**
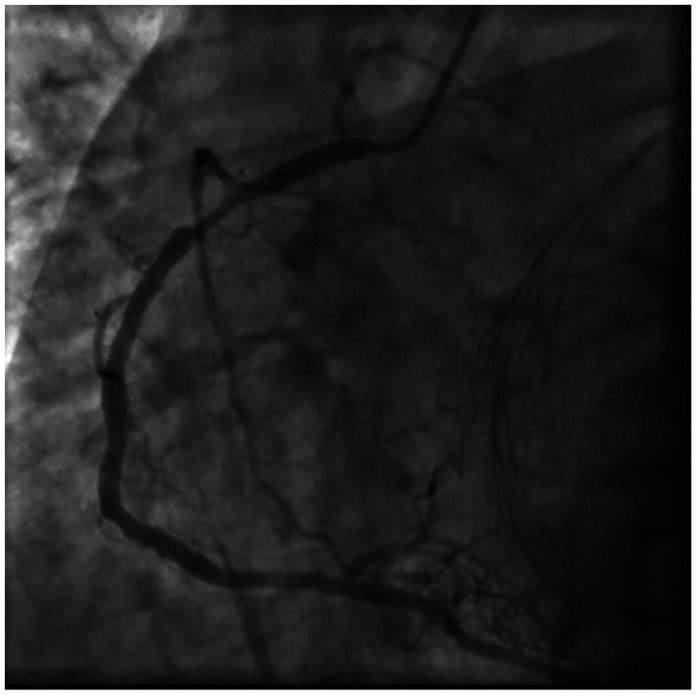
Coronary angiography view of RCA proximal 90% stenosis.

A 55-year-old male patient was referred to our transplantation clinic. The patient was on a 3/7 dialysis program. During his cardiac assessment, electrocardiogram (ECG) findings were non-significant, and his left ventricular ejection fraction (LVEF) was 58%. He was on colchicine treatment for mild pleural and pericardial effusion. Myocardial perfusion scintigraphy (MPS) revealed anterior and apical non-reversible and reversible perfusion defects. Three-vessel CAD was diagnosed by coronary angiography (CAG) (Figures 1, 2). An off-pump CABGx3 [the left internal mammary artery to the left anterior descending artery (LIMA-LAD), aort to right posterior descending artery (Ao-RPD), Ao to second optuse marginal branch of circumflex artery (Ao-CxOM2)] was performed without the use of blood products. The patient's pre-CABG laboratory values were as follows: hemoglobin (Hgb) 8.5 g/dl, hematocrit (Hct) 24.6%, platelets (PLT) 172,000/μl, and creatinine (Cr) 6.67 mg/dl. Post-CABG, the following values were measured: Hgb 7.61 g/dl, Hct 22.8%, PLT 146,000/μl, and Cr 9 mg/dl. On post-CABG day 5, the patient was transferred to nephrology while continuing oral cardiac treatment.

The patient was subsequently hospitalized at an external center due to decompensation and pancytopenia. During the etiological investigation, potential causes such as marrow suppression, nutritional deficiencies, and drug reactions were explored. Colchicine-related pancytopenia was also considered. After discontinuation of colchicine, the pancytopenia resolved. After a 1-month hospital stay involving intensive hemodialysis and close hematological follow-up, preparations for RT began. Serological tests were performed, and panel reactive antibodies (PRA) were in normal range. Successful RT from a living donor was performed on the 60th day post-CABG. The immunosuppressive and prophylactic treatment protocols in our RT clinic are as follows: intravenous prednisolone administration started with 1,000 mg on the preoperative day, 500 mg intra-operatively, 250 mg on post-operative day (POD) 1, 125 mg on POD 2, 100 mg on POD 3, 80 mg on POD 4, 60 mg on POD 5, and 40 mg on POD 6. Following discharge, oral prednisolone was prescribed at 20 mg daily for the first month, 15 mg daily for the second month, 10 mg daily for the third month, and then maintained lifelong at 5 mg daily.

In addition to steroid therapy, doses of tacrolimus and mycophenolate sodium were adjusted based on tacrolimus level monitoring. Due to the recurrence of neutropenia in the third month following RT, mycophenolate sodium was discontinued, and everolimus therapy was initiated, with dosing guided by serum level monitoring.

At the 12-month follow-up, the patient was found to be both cardiac and renal stable.

## Case 2

A 58-year-old male with hypertension and secondary diabetes due to previous steroid treatment in his medical history was referred for cardiac evaluation while preparing for RT. He had been diagnosed with focal segmental glomerulosclerosis (FSGS) 3 years prior and treated with steroids for 5 months. Transthoracic echocardiography (TTE) and ECG findings were non-significant. CAG revealed three-vessel CAD. An off-pump CABGx3 [the right internal mammary artery to LAD (RIMA-LAD), LIMA to first diagonal and optuse marginal branch (LIMAD1-OM in a saphenous Y graft configuration)] was performed because of the porcelain aorta without blood transfusion. Pre-CABG laboratory values were as follows: Hgb 16.3 g/dl, Hct 47.6%, PLT 209,000/μl, and Cr 5.15 mg/dl. Post-CABG laboratory values were Hgb 12.5 g/dl, Hct 38%, PLT 166,000/μl, and Cr 6.33. Perioperative routine prophylaxis with valganciclovir 450 mg 1 × 1 p.o. and sulfamethoxazole/trimethoprim 80/400 mg 1 × 1 p.o. was applied.

There was no significant difference in post-CABG TTE. On post-CABG day 5, the patient was discharged with the following treatment regimen: acetylsalicylic acid 100 mg once daily, metoprolol 50 mg once daily, and lercanidipine 10 mg once daily.

The patient was placed on the 2/7 hemodialysis program after CABG due to worsening kidney function. Creatinine clearance, 24-h proteinuria, ultrasound imaging of the kidneys, and serological tests were re-evaluated. PRA levels were within the normal range. Successful RT from a living donor was performed on the 38th day post-CABG. Immediate graft function was achieved with adequate urine output post-surgery. Immunosuppressive treatment was arranged in accordance with the protocols of our transplant team. Three months of trimethoprim-sulfamethoxazole (TMP-SMX) 480 mg daily against *Pneumocystis jirovecii* and valganciclovir 450 mg daily against cytomegalovirus (CMV) were prescribed as prophylaxis.

At 12-month follow-up, the patient was found to be both cardiac and renal stable.

## Discussion

Patients with ESRD have a higher risk of cardiovascular disease compared to the general population, and cardiac surgery carries a higher risk. Moreover, CAD and heart surgery can negatively affect the early and late outcomes of RT.^1^

CABG remains a cornerstone in the management of multi-vessel CAD (5). It offers superior revascularization in cases of multi-vessel CAD due to its ability to bypass complex and diffusely calcified lesions, which are common in ESRD and RT populations. Meta-analyses have consistently shown that CABG reduces the risk of major adverse cardiac and cerebrovascular events (MACCEs), including myocardial infarction and repeat revascularization, compared to PCI. For our young RT recipients, this durability is especially critical given their longer expected lifespan compared to dialysis-dependent patients (6–8). Advances in surgical techniques have resulted in two principal approaches: OPCAB, which is performed on a beating heart without the use of cardiopulmonary bypass (CPB), and ONCAB, which employs CPB and cardioplegic arrest. Each method has distinct advantages and limitations, often tailored to the patient's comorbidities and anatomy and surgeon’s expertise.

OPCAB procedures, similar to ONCAB, were performed under general anesthesia via median sternotomy. Stabilization of the target vessels was accomplished with the Acrobat-i stabilizer (Getinge). Hemodynamic stability during heart positioning for distal anastomosis was maintained using the Trendelenburg position and pharmacologic management with intravenous noradrenaline infusion as needed. Anticoagulation was achieved by administering intravenous heparin at 1.5 mg/kg of body weight, ensuring an activated clotting time (ACT) above 300 s throughout the procedure. To improve surgical field visibility, proximal occlusion of the target vessel was performed with a micro-vessel occluder, and a sterile medical air blower was used to clear blood from the area. The distal artery was routinely kept open, and neither ischemic preconditioning nor intracoronary shunts were used. Whenever possible, the LAD was grafted first to restore blood flow to the anterior wall as early as possible (9). Anastomoses to the right coronary artery (RCA) were avoided unless critical stenosis (>90%) was present. Unless there is a specific indication, we do not perform thoracic CT prior to CAB surgery. Of course, epiaortic ultrasound evaluation of the ascending aorta is more effective than palpation by the surgeon. However, in our second case, the porcelain aorta was so evident by palpation that, given our extensive experience with OPCAB, palpation alone was sufficient.

Multiple studies, including meta-analyses, indicate comparable 10-year survival rates between OPCAB and ONCAB. In addition, other long-term endpoints, including angina, graft patency, heart failure, rehospitalization, and stroke, suggest similar adverse events (10). A recent study involving 24,883 participants showed that the benefit of OPCAB on mortality at 8-year follow-up did not differ between diabetic and non-diabetic patients (11). On the other hand, another recent study found ONCAB superior to OFCAB due to shorter procedure time and fewer MACCEs (9, 12). These controversial results indicate that the operator's expertise plays a critical role, particularly for OPCAB, where surgeon skill significantly influences graft patency and overall outcomes.

OPCAB offers several advantages for ESRD patients awaiting RT. By avoiding CPB, OPCAB reduces the risk of systemic inflammation, coagulopathy, and the need for blood transfusions, thereby minimizing immunological challenges that could trigger graft rejection (13). Studies have highlighted the importance of minimizing blood transfusions to prevent immune system activation and potential graft rejection (14, 15). In addition, preoperative measurement of PRA levels is crucial for assessing the risk of graft rejection and tailoring immunosuppressive therapy (16). OPCAB also provides an advantage as a less invasive option in patients with severe aortic calcification, as in case 2.

The most recent propensity-match analysis comparing OPCAB and ONCAB in ESRD patients not on dialysis showed no differences in mortality, stroke, and post-operative dialysis rates. On the other hand, longer hospital stays and re-operation due to bleeding/tamponade were reported more frequently in ONCAB (17). In patients with chronic kidney disease (CKD), even in the absence of RT, the frequent occurrence of comorbidities such as porcelain aorta (as seen in case 2), increased frailty, and polypharmacy make them more susceptible to drug side effects (as seen in case 1) and hematological complications. Consequently, OPCAB demonstrates significantly better outcomes than ONCAB in terms of short-term mortality (18).

In our practice, we prioritize the use of arterial grafts in high-risk patients with multiple comorbidities, aligning with recent studies demonstrating better clinical outcomes for patients with ESRD undergoing CABG when arterial grafts are used instead of saphenous vein grafts (19). For instance, the RAPCO trials reported a 29% reduction in MACCEs over 15 years when using radial artery grafts instead of saphenous vein grafts (20). However, in patients with ESRD, considering that dialysis and an AV fistula may still be required even after RT, we avoid using the radial approach in CAG and similarly refrain from using radial grafts in CABG.

The appropriate approach for patients with CAD who were scheduled for RT remains a topic of debate. Stabilizing coronary flow as a first step reduces the risk of myocardial ischemia during subsequent transplantation. This stabilization allows the heart to better cope with the increased hemodynamic burden of major surgery and intraoperative cardiac stress. For instance, pancytopenia following combined surgery, as seen in case 1, causes significant risks in terms of both graft patency and cardiac stability. Furthermore, it may necessitate undesirable modifications to immunosuppressive treatment regimens.

Managing two major surgeries simultaneously is associated with prolonged surgery duration and increased anesthesia exposure, raising challenges in the stabilization of blood pressure and intraoperative fluid balance, especially in ESRD patients. The interval between CABG and RT can optimize the patient's immunological status and reduce the risk of graft rejection. On the other hand, in the combined strategy, immunosuppressive therapy management may be more complex, potentially endangering both cardiac and renal functions. In addition, in this fragile patient group, as in the first case report, if a complication develops between two major surgeries, transplantation can be postponed until the patient is stable.

This case series is unique in presenting patients who underwent OPCAB followed by RT without blood transfusion, coupled with a 12-month follow-up period. By staging the procedures, we planned to reduce the risk of warm ischemia in the kidney, which may be caused by hypotension and prolonged surgery duration during a combined CABG and RT procedure. We present a different perspective suggesting that a staged approach may enable better management of complications in this patient group characterized by multiple comorbidities and polypharmacy. This approach may help preserve the viability of the graft, which is particularly valuable when obtained from a living donor.

A multidisciplinary team involving cardiology, cardiothoracic surgery, and transplant nephrology assessed both patients and determined that they were poor candidates for percutaneous coronary intervention (PCI) due to complex CAD, low hemoglobin levels, and a hypercoagulable state; consequently, decided on staged surgery. In the pre-RT cardiac evaluation of patients with ESRD, whose exercise capacity is already limited by many other accompanying chronic diseases, clinical suspicion by cardiology and its tests are diagnostic. The comprehensive approach provided by transplant nephrology ensures that patients have an uninterrupted follow-up process. Alongside an experienced clinical and surgical team, perhaps the most crucial point is to include the patient in this multidisciplinary decision-making process. We informed our patients comprehensively about both combined and staged surgery options and discussed the advantages and disadvantages of each approach.

## Conclusion

Off-pump CABG followed by staged RT presents a beneficial approach for patients with ESRD and significant CAD. Common comorbidities in these patient groups, such as porcelain aorta (as seen in case 2), increased frailty, polypharmacy, and hematological complications, highlight the advantages of OFCAB. In the current era, where major surgeries performed in the same session are increasingly popular, this strategy provides a perspective that can prepare patients for a more successful RT and post-operative follow-up by ensuring immunological integrity and the complete wellbeing of patients. Each patient is unique, and the decision-making process should be individualized.

## Limitations

This study has some significant limitations. Since it includes only two patients, the results cannot be applied to larger populations. In addition, the outcomes may be affected by the high level of experience of the transplant and surgical teams, as well as the specific facilities and resources available at our hospital, which may not be the same in other centers. Another limitation is that we did not routinely perform preoperative thoracic CT scans before CABG unless there was a clear indication. Although epiaortic ultrasound evaluation of the ascending aorta usually provides reliable information, in the second case, the porcelain aorta was so obvious by palpation that imaging was deemed unnecessary due to the team's significant OPCAB experience. However, in less experienced centers, imaging may be needed for better surgical planning. These points indicate that surgical strategies should be adapted based on the team's experience and hospital resources.

## Data Availability

The original contributions presented in the study are included in the article/Supplementary Material, further inquiries can be directed to the corresponding author.

## Footnotes

^1^Patients with end-stage renal disease have a higher risk of cardiovascular disease compared to the general population and cardiac surgery is very high risk. Moreover, cardiovascular diseases and heart surgery negatively affect the early and late results of kidney transplantation.
